# Characterization of Polyethylene Using a New Test Method Based on Stress Response to Relaxation and Recovery

**DOI:** 10.3390/polym14142763

**Published:** 2022-07-06

**Authors:** Furui Shi, P.-Y. Ben Jar

**Affiliations:** Department of Mechanical Engineering, University of Alberta, 10-203 Donadeo Innovation Centre for Engineering, 9211-116 Street NW, Edmonton, AB T6G 1H9, Canada; ben.jar@ualberta.ca

**Keywords:** multi-relaxation-recovery test, deformation mechanism, polyethylene, activation energy, modeling

## Abstract

A novel multi-relaxation-recovery (RR) test was proposed based on cyclic stages of stress relaxation and stress recovery. Three nonlinear visco-elastic models, that is, the standard model and two models with two dashpots connected either in parallel or in series, were examined for the analysis of the test results. Each model contains a time-dependent, viscous branch and a time-independent, quasi-static branch. The examination suggests that the standard model can determine the long-term, load-carrying performance of polyethylene (PE) and identify a transition point for the onset of plastic deformation in the crystalline phase, but the models with two dashpots connected either in parallel or in series are needed to provide a close simulation of the experimentally measured stress response in both relaxation and recovery stages of the RR test. In this work, the mechanical performance of two PEs was compared based on RR test results at room temperature. The RR tests were also conducted at elevated temperatures to explore the possibility of quantifying the activation energies for deformation of the dashpots at the relaxation stage. It was found the RR test has the advantage of separating the time-dependent and time-independent components of stiffness of the materials. The study concludes that the RR test can provide data for determining parameters in Eyring’s model in order to characterize the contribution of time-dependent and time-independent components of the stress response to PE’s deformation.

## 1. Introduction

Semi-crystalline polymers (SCPs) have been increasingly used in industrial applications due to their potential for fulfilling the performance requirements, with the advantages of chemical resistance and installation flexibility [1,2,3]. As the most studied SCP, polyethylene (PE) has a global demand of nearly a hundred million metric tons in 2018, equivalent to approximately US$164 billion, with an annual growth of 4.0% [4,5]. With the significant improvement in PE’s performance, its applications to engineering structures have steadily increased in recent years [6,7]. A total of 95% of the plastic pipes in the United States are PE pipes [8]. PE pipes are increasingly applied to water and gas transportation [9]. However, PE’s failure can also cause tremendous economic losses and sometimes fatalities [10,11,12]. Therefore, urgent attention is needed for the proper evaluation of PE’s performance. As a result, literature has shown tremendous work in experimental testing and performance modeling for the characterization of PE’s mechanical behavior [13,14,15]. Currently, some bottlenecks still exist, especially in linking its mechanical performance with the dominant deformation mechanisms. These bottlenecks are known to be caused by several issues. Firstly, many models used for the analysis of the mechanical test results require assumptions that are practically unrealistic. For example, characteristic relaxation time for the viscous deformation has often been assumed to be constant, independent of the deformation level or of the material [16,17,18]. Secondly, modeling based on spring and dashpot elements often assumed that the viscous stress component is a function of total strain rate, rather than the strain rate across the dashpot element [16]. Maxwell and Voigt-Kelvin models are the basic models that use spring and dashpot to simulate the viscous deformation [19,20], with the assumption of a linear relationship between viscous stress and strain rate across the dashpot. However, these linear models are insufficient to describe the complex, nonlinear stress response of PE to deformation [19,21], Drozdov et al. have proposed a model with 15 parameters to simulate the nonlinear deformation behavior, but the model was only used for the analysis prior to the yield point [22]. Alternatively, Anand and coworkers developed a thermo-mechanical-coupled theory with more than thirty parameters to mimic the large deformation, but this theory was only applicable to amorphous polymers [23]. Some other models that consider nonlinear constitutive equations are only applicable to a specific loading mode. For example, Boyce et al. proposed a constitutive model to simulate the loading behavior of poly(ethylene terephthalate), but failed to predict correctly the unloading behavior [24]. Mirkhalaf et al. modeled the post-yield response of amorphous polymers, also without the validation of the unloading behavior [25]. Models in the literature that considered the unloading showed that relaxation and recovery behaviors could not be simulated using the same model parameters. For example, Detrez et al. characterized SCPs for loading, relaxation, and unloading behaviors, but failed to simulate the recovery behavior after the unloading [26].

In this paper, a new mechanical test, named the multi-relaxation-recovery (RR) test, is proposed to separate the quasi-static stress response to deformation from the viscous counterpart. As suggested by the name, the RR test consists of multiple stages of stress relaxation and recovery and the associated loading and unloading. These stages are repeated during the RR test to characterize the quasi-static and the viscous stress responses to deformation, based on which spring-dashpot models are examined to identify the proper model parameters that can simulate the experimentally-determined stress-deformation curve. Specifically, this paper considers three models which are the standard model [27], the model with two dashpots connected either in parallel (to be named the Parallel model hereafter), or in series (to be named the Series model), all of which are based on the Eyring’s law for the stress response of the dashpot [28,29,30]. Each of these models consists of two branches, one being a viscous branch to simulate the time-dependent stress response to deformation, and the other a quasi-static branch to simulate the time-independent stress response. This paper provides details of the RR test and the analysis for determining parameter values for all spring and dashpot elements in the models. The analysis also examines the possibility of using the RR test results to identify the critical strokes for the onset of deformation transition that has been reported in the previous work [17,31]. Furthermore, two case studies are presented using the RR test. One is to compare two types of high-density polyethylene (HDPE) for their mechanical performance, and the other to use RR test results at different temperatures to determine the activation energies for deformation during the stress relaxation.

## 2. RR Test

Tests that consist of multiple deformation stages have been developed to characterize the time- and strain rate-dependent deformation behaviors of polymers [16,17,31]. In the work that is concerned about the mechanical performance, analysis of the test data are often based on a constant characteristic relaxation time, i.e., independent of deformation level or material [17,31,32].

**Figure 1:**
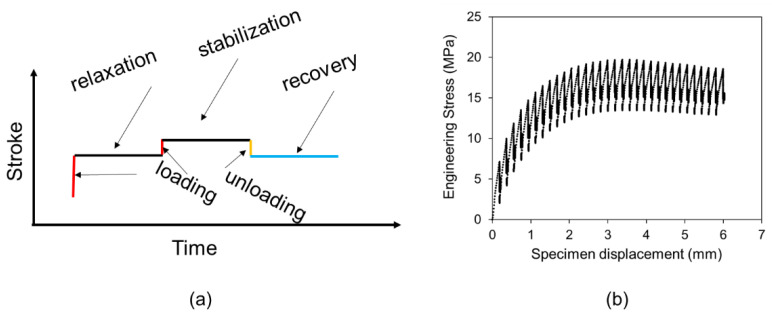
Multi-relaxation-recovery test: (**a**) stroke versus time in one stage of the RR test; (**b**) a sample curve showing the engineering stress versus displacement (taken from data for HDPE-a).

The idea for the RR test described in this paper is to address the above deficiency, that is, to collect stress response at both stress relaxation and stress recovery modes and without the assumption of constant characteristic relaxation time. As shown in Figure 1a, each stage of the RR test contains four test modes, including loading, relaxation, unloading, and recovery. Note that stress relaxation is introduced twice, one being labeled as relaxation in Figure 1a and the other ‘stabilization.’ The latter is to stabilize the deformation process before the specimen is unloaded for stress recovery. Loading between relaxation and stabilization is through a much smaller displacement increment than the loading between recovery and the following relaxation. In this study, displacement increment for the former is about one-fifth of that for the latter.

The deformation stage shown in Figure 1a is repeated cyclically to cover a wide range of deformation levels, which could continue till the specimen fractures. However, for the work reported here, the RR tests were terminated at the point where necking became noticeable to the naked eyes, in order to reduce the amount of data for the analysis in view that the test is in the development stage. A sample curve of engineering stress versus displacement, collected from an RR test, is shown in Figure 1b.

**Figure 2:**
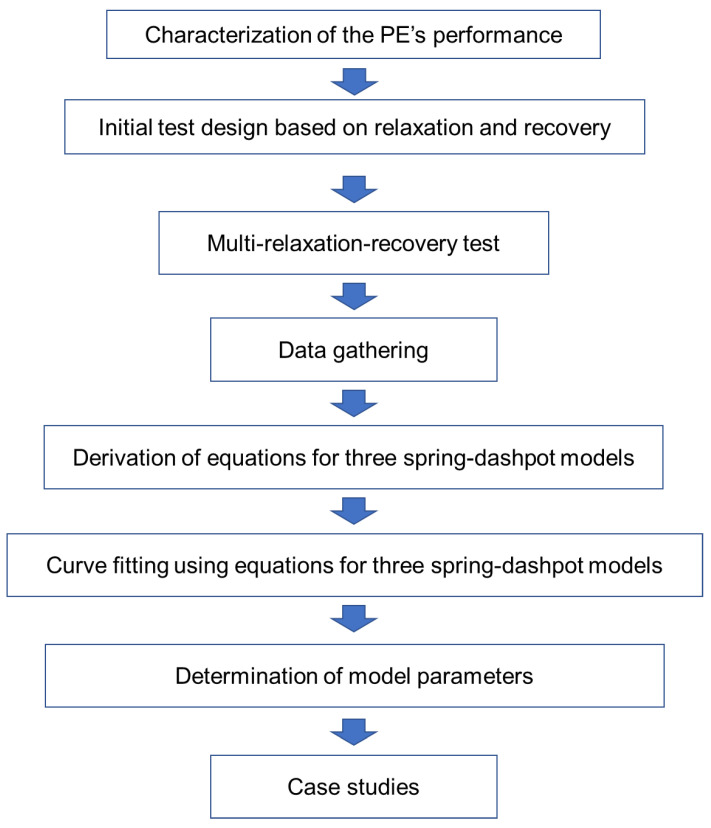
Flow chart of the methodological procedure in this study.

Three spring-dashpot models, including the standard model [6], the Parallel model, also considered in Refs. [33,34,35,36], and the Series model, were applied to simulate the relaxation and recovery behavior in the RR test, to extract the model parameters by fitting the experimentally determined engineering stress-displacement curves. Each of these models consists of two branches, one being a viscous branch to simulate the time-dependent stress response to deformation, and the other a quasi-static branch to simulate the time-independent stress response. Quasi-static stress ($\sigma_{\mathrm{qs}}$) was obtained as a function of stroke by an approach, named combined relaxation-recovery (CRR) approach here, which is based on a widely accepted concept that a common $\sigma_{\mathrm{qs}}$ exists for relaxation and recovery phases at the same specimen displacement. Compared to the analysis of multi-relaxation tests in the literature, the CRR approach allows the variation of characteristic relaxation time ($\tau_{v}$) with deformation, and thus removes the assumption of a constant $\tau_{v}$ that has been used in the past [17,31,32]. RR test provided a data set for the determination of model parameters, which was then applied to the characterization of the time-dependent and time-independent performance of polymers. In this study, using the results, mechanical performance for two types of high-density polyethylene (HDPE) was compared. In addition, the RR test results at elevated temperatures were used to obtain the activation energies. The methodological procedure of this study is shown in the flow chart, as shown in Figure 2.

## 3. Analysis of RR Test Results Based on Spring-Dashpot Models

**Figure 3:**
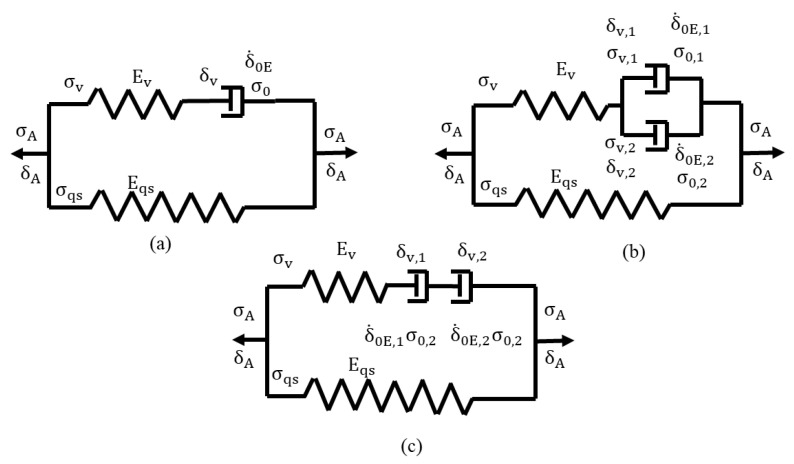
Schematic diagrams of three models used for the data analysis: (**a**) the standard model; (**b**) the Parallel model; (**c**) the Series model.

The three spring-dashpot models considered for the data analysis are depicted in Figure 3, namely, the standard model [17], the Parallel model [33,34,35,36], and the Series model [37]. For each model, the lower branch in Figure 3 represents the $\sigma_{\mathrm{qs}}$ response to deformation and the upper branch the viscous counterpart. Since the two branches are connected in parallel, total specimen displacement is equivalent between the two branches, so is the total stroke rate. Note that displacement and the stroke change measured by the test machine follow a one-to-one relationship. Therefore, displacement is defined as the stroke change of the test machine. For the Parallel model, the equivalence is also applicable to the stroke and the stroke rate for the two dashpots. For the Series model, on the other hand, stress applied to the two dashpots is equivalent, so is the stress rate, but the stroke and the stroke rate for the two dashpots could be different.

### 3.1. Standard Model

For the standard model in Figure 3a, the applied stress is represented by $\;\sigma_{A}$, the applied stroke $\delta_{A}$, the viscous stress component $\sigma_{v}$, the spring constant in the viscous branch $E_{v}$, the spring constant in the quasi-static branch $E_{\mathrm{qs}}$, and the quasi-static stress $\sigma_{\mathrm{qs}}$. Halsey et al. originally proposed Eyring’s process for deformation of a polymer [38], and this theory is widely accepted [39,40]. Eyring’s equation, as shown below, is adopted to govern the stroke rate (${\dot{\delta }}_{v}$) of the dashpot:

(1) $${\dot{\delta }}_{v}={\dot{\delta }}_{0E}\;\sin h\left(\sigma_{v}\;/{\;\sigma }_{0}\;\right)$$

where ${\dot{\delta }}_{0E}$ and $\sigma_{0}$ are the reference stroke rate and the reference stress, respectively, of the dashpot. The relationship between the stroke rate of the spring in the viscous branch $\left({\dot{\delta }}_{E_{v}}\right)$ and the corresponding stress rate $\left({\dot{\sigma }}_{v}\right)$ is given below.

(2) $${\dot{\delta }}_{E_{v}}={\dot{\sigma }}_{v}\;/{\;E}_{v}$$

Based on Equations (1) and (2), the applied stroke rate for the upper branch, which is equivalent to ${\dot{\delta }}_{A}$, can be expressed as

(3) $${\dot{\delta }}_{A}={\dot{\sigma }}_{v}\;/{\;E}_{v}+{\dot{\delta }}_{0E}\;\sin h\left(\sigma_{v}\;/{\;\sigma }_{0}\;\right)$$

In the mode of stress relaxation or stress recovery at constant $\delta_{A}$, Equation (3) becomes

(4) $$\sigma_{v}=2\sigma_{0}{\tan h}^{-1}\left\{\tan h\left[\sigma_{v}\left(0\right)\;/\;2\sigma_{0}\right]\exp\left(-t\;/{\;\tau }_{v}\right)\right\}$$

where t is the time measured from the beginning of stress relaxation or stress recovery, $\sigma_{v}\left(0\right){\;\mathrm{is}\;\sigma }_{v}$ at t = 0, and $\tau_{v}$ the characteristic time for stress relaxation or stress recovery, which is a combined parameter of $\sigma_{0}$, $E_{v}$ and ${\dot{\delta }}_{0E}$:

(5) $$\tau_{v}={\;\sigma }_{0}\;/\;\left(E_{v}{\dot{\delta }}_{0E}\right)$$

Since $\sigma_{A}$ is the sum of stresses applied to the viscous and quasi-static branches, $\sigma_{v}$ and $\sigma_{\mathrm{qs}}$, $\sigma_{A}$ for the standard model can be expressed as

(6) $$\sigma_{A}={\;\sigma }_{\mathrm{qs}}+2\sigma_{0}{\tan h}^{-1}\left\{\tan h\left[\sigma_{v}\left(0\right)\;/\;2\sigma_{0}\right]\exp\left(-t\;/{\;\tau }_{v}\right)\right\}$$

In this study, Equation (6) is used to examine the suitability of the standard model for reproducing the RR test results.

### 3.2. Parallel Model

For the Parallel model in Figure 3b, ${\dot{\delta }}_{v,1}$, ${\dot{\delta }}_{0E,1}$, $\sigma_{0,1}$ represent stroke rate, reference stroke rate, and reference stress, respectively, of dashpot 1, and the corresponding terms with subscript 2, i.e., ${\dot{\delta }}_{v,2}$, ${\dot{\delta }}_{0E,2}$, and $\sigma_{0,2}$ for dashpot 2. Again, Equation (1) is adopted to govern the stroke rate for each dashpot. Here, process 1 is used to represent the process with a larger $\tau_{v}$ value and process 2 the process with a smaller $\tau_{v}$ value. As mentioned earlier, since the two dashpots are connected in parallel, the two dashpots have the same stroke and stroke rate, and the expression for the stroke rate applied to the dashpots is given below.

(7) $${\dot{\delta }}_{v,1}={\dot{\delta }}_{v,2}={\dot{\delta }}_{A}-{\dot{\sigma }}_{v}\;/\;E_{v}$$

Since stresses of the two dashpots are additive, based on Equation (1), total stress of the viscous branch is

(8) $$\begin{array}{cc} {\;\sigma }_{v} & =\sigma_{A}-\sigma_{\mathrm{qs}}\\ & =\sigma_{0,1}\;\mathrm{asinh}\left[\left({\dot{\delta }}_{A}-{\;\dot{\sigma }}_{v}\;/{\;E}_{v}\right)\;/{\dot{\delta }}_{0E,1}\right]+{\;\sigma }_{0,2}\;\mathrm{asinh}\left[\left({\dot{\delta }}_{A}-{\dot{\sigma }}_{v}\;/{\;E}_{v}\right)\;/{\dot{\delta }}_{0E,2}\right]\; \end{array}$$

### 3.3. Series Model

For the Series model, in Figure 3c, the total deformation of the viscous branch (equivalent to $\delta_{A}$) is equal to the summation of deformations of the spring in the branch and the two dashpots. Same as the other two models, Equation (1) is adopted to govern the stroke rate for the two dashpots, as a function of the stress applied to the viscous branch ($\sigma_{v}$). Therefore, the stress rate applied to the viscous branch (${\;\dot{\sigma }}_{v}$) can be expressed using the following expression.

(9) $${\dot{\sigma }}_{v}={\;E}_{v}\left[{\dot{\delta }}_{A}-{\dot{\delta }}_{0E,1}\;\sin h\left(\sigma_{v}\;/\;\sigma_{0,1}\right)-{\dot{\delta }}_{0E,2}\;\sin h\left(\sigma_{v}\;/{\;\sigma }_{0,2}\right)\right]$$

Equations (6), (8) and (9) for the three models were solved by curve fitting, and the details are given in Section 5.

## 4. Experimental Details of the RR Test Used in the Study

### 4.1. Materials and Specimen Dimensions

Two types of HDPE were used in the study. One is qualified as a PE100 resin (HDPE-a) and the other not qualified (HDPE-b). Their characteristics are listed in Table 1, provided by the material suppliers.

**Figure 4:**
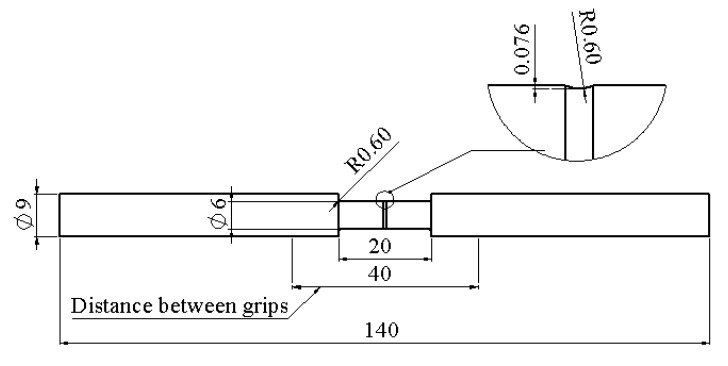
Geometry and dimensions of specimens used for the RR tests.

Specimens used for the RR test had an axisymmetric geometry, as shown in Figure 4, with the same dimensions as the specimens used previously in our study [41]. That is, the overall length is 140 mm, and the length and diameter in the gauge section are 20 and 6 mm, respectively. Same as before, the specimens contained a small circumferential groove in the middle of the gauge section, with a groove depth of 0.076 mm, to ensure that necking started there. Each specimen was gripped using a 50-mm-long steel tab at both ends to avoid slippage during the RR test.

### 4.2. Test Conditions

A computer-controlled universal test machine (Qualitest Quasar100, Lauderdale, FL, USA) was used to conduct the RR tests. The test program was designed to have each stage follow the scheme depicted in Figure 1a as a function of time. For this study, a loading period of 12 s was used to reach the relaxation phase, and a period of 10,000 s for stress relaxation at a fixed stroke. Note that at the end of the relaxation phase, a loading period of 3 s was given before the stabilization phase which was also for a period of 10,000 s. At the end of the stabilization phase, the specimen was unloaded for 3 s before the recovery phase for a period of 10,000 s as well. Crosshead speed for all loading and unloading phases was set to be 1 mm/min. However, a period of about 1.5 s was needed for the crosshead to reach the specified speed. Therefore, the actual stroke increment for loading was about 0.2 mm between the recovery and the next relaxation phases, and the stroke change of about 0.033 mm between the relaxation and stabilization phases (loading) and between stabilization and recovery phases (unloading). In total, about 30 cycles of the loading scheme shown in Figure 1a were applied to the specimen, to introduce a total specimen displacement, in terms of stroke of the test machine, of around 6 mm.

Repeated RR tests were conducted at room temperature using at least two specimens for each HDPE, to ensure consistency of the reproducibility as that obtained previously [17,41]. RR tests were also conducted at elevated temperatures of 313, 318, 328, 343, 358, and 368 K, to determine the activation energies, but only for HDPE-a of one specimen at each temperature. The use of one specimen at each temperature was mainly because of the good reproducibility of the test results and a long period of about 11 days required for each RR test. Besides, each RR test provides 30 sets of data for the analysis, with each set including loading, relaxation, unloading, and recovery. Therefore, the limited number of tests at elevated temperatures have actually provided more than 180 sets of data for the analysis.

## 5. Results and Discussion

**Figure 5:**
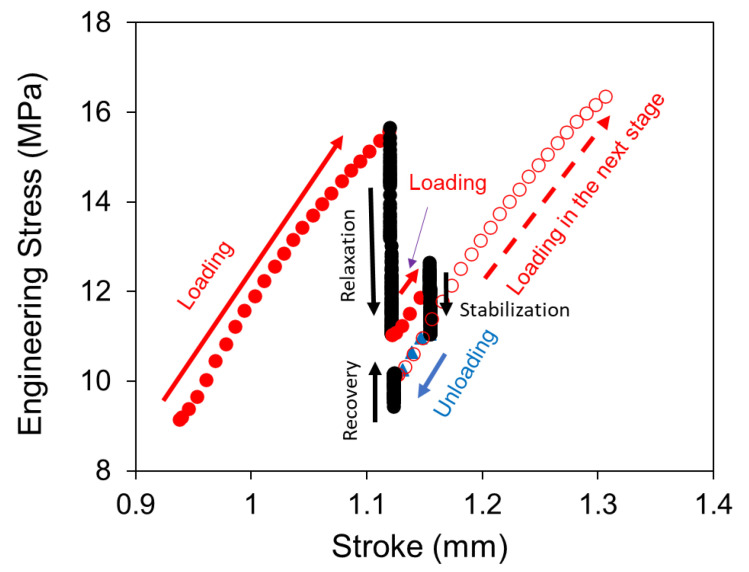
A sample curve of engineering stress versus stroke, from one cycle of the RR test on HDPE-a at room temperature.

**Figure 6:**
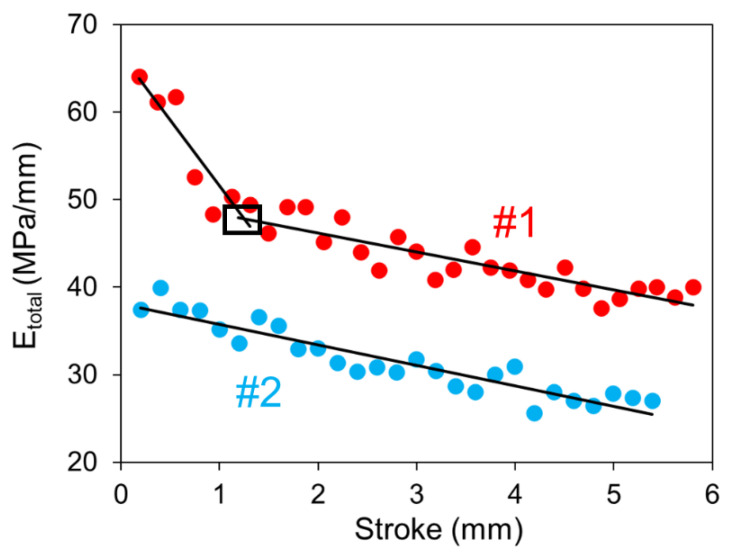
Total stiffness ($E_{\mathrm{total}}$) versus stroke for HDPE-a and HDPE-b at room temperature.

A typical engineering stress-stroke curve for one cycle of the RR test is shown in Figure 5, as complementary to the stroke-time plot in Figure 1a. Figure 5 indicates that a commonly observed hysteresis loop from loading-unloading of polymeric materials [42] is hardly visible between unloading before the recovery phase and the initial loading after the recovery phase. Overlap of the unloading curve to the recovery phase and the initial loading curve to the following relaxation phase suggests that the specimen was in a nearly fully relaxed state after the stabilization. The slope for this part of the stress-stroke curve is presented in Figure 6 as a function of stroke for the two HDPEs [43], representing the total stiffness $\left(E_{\mathrm{total}}\right)$ of the material in the fully relaxed state. The figure indicates that an early, relatively fast rate of $E_{\mathrm{total}}$ drop occurred in HDPE-a. For HDPE-b, the $E_{\mathrm{total}}$ drop has a constant rate which is close to the drop rate of $E_{\mathrm{total}}$ for HDPE-a at the large stroke.

Three models, as described in Section 3, were used to analyze the RR test results. For each model, the CRR approach was used to determine values for the model parameters, based on the assumption that $\sigma_{\mathrm{qs}}$ is only a function of specimen displacement (in terms of stroke of the test machine). This assumption is consistent with the common belief that relaxation and recovery processes at a given stroke should eventually reach the same stress level [44]. In the CRR approach, a $\sigma_{\mathrm{qs}}$ value was firstly selected within the stress range between the end of the relaxation phase and the end of the following recovery phase. Values for parameters in each of the three models in Figure 3 were then searched to provide the best fit to the experimental curve in the relaxation and recovery phases. A two-folded fitting criterion was applied to determine the most suitable $\sigma_{\mathrm{qs}}$ value. One was the number of experimental data points in a given marker size passed by the model-generated curve, and the other the consistency of the overall trend of the model-generated curve with the trend shown by the experimental data.

In this study, $\sigma_{\mathrm{qs}}$ was determined using the standard model based on Equation (6), with $\sigma_{v}\left(0\right)$ being the difference between $\sigma_{A}$ and $\sigma_{\mathrm{qs}}$ at the beginning of the relaxation or the recovery phase. The $\sigma_{\mathrm{qs}}$ values were then applied to the other two models to determine their model parameters.

Two case studies are presented below to demonstrate results from the CRR approach. The first case study is to compare the quality of the curve fitting generated by the three models, and to investigate the pros and cons of the three models for mimicking the experimentally-obtained curves. The second case study is to explore the possibility of using the RR tests at elevated temperatures to determine the activation energies for Eyring’s model. Note that both HDPE-a and HDPE-b were used in the first case study, but for the second case study, only HDPE-a was used. Determination of the activation energies in the second case study was based on the Parallel model because results from the first case study have indicated that among the three models considered here, the Parallel model is most suitable for mimicking the stress response to deformation in the RR test.

### 5.1. Case Study 1: Comparison of Three Models Depicted Above

The simulation of relaxation and recovery using the standard model can be completed using Equation (6). Curve fitting was performed by firstly choosing a $\sigma_{\mathrm{qs}}$ value, and then adjusting the $\sigma_{0}$ and $\tau_{v}$ values for the standard model to fit the experimentally obtained stress-time curves from the relaxation and recovery phases. Note that for the five unknowns at a given stroke, i.e., $\sigma_{\mathrm{qs}}$ and the two sets of $\sigma_{0}$ and $\tau_{v}$ (one set for the relaxation phase and the other set for the recovery phase), were the values that could provide the best fit to the experimental data obtained from the two phases. This fitting procedure was repeated through the whole RR tests to establish the variation of values for the five parameters as a function of stroke.

The simulation of relaxation and recovery using the Parallel and the Series models was performed using Equations (8) and (9), respectively. It was assumed that $\sigma_{\mathrm{qs}}$ values used for the Parallel and the Series models are the same as those determined from the standard model. Since ${\dot{\delta }}_{A}$ is 0 in the relaxation and recovery phases, there were four unknowns in each of Equations (8) and (9), to be determined from the curve fitting: $\sigma_{0,1}$, $\sigma_{0,2}$, $E_{v}{\dot{\delta }}_{0E,1}$, and $E_{v}{\dot{\delta }}_{0E,2}$. Equations (8) and (9) are ordinary differential equations involving the unknown function $\sigma_{v}\left(t\right)$ and its derivatives with respect to time t. Matlab was applied to solve these ordinary differential equations. To solve these ordinary differential equations using Matlab, function “Ode15i” in Matlab was used to determine these four parameters in the Parallel model, based on Equation (8), as this equation is a fully implicit differential equation which can be solved using “Ode15i” [45]. For the Series model, on the other hand, the four parameters were determined using the function “Ode45” in Matlab, as this function is designed to solve a nonstiff differential equation like Equation (9) [46]. As mentioned earlier, the model-generated curves were checked by naked eyes to ensure that the fitting criterion of passing through as many experimental data points as possible and showing the same trend as the experimental curves were met.

**Figure 7:**
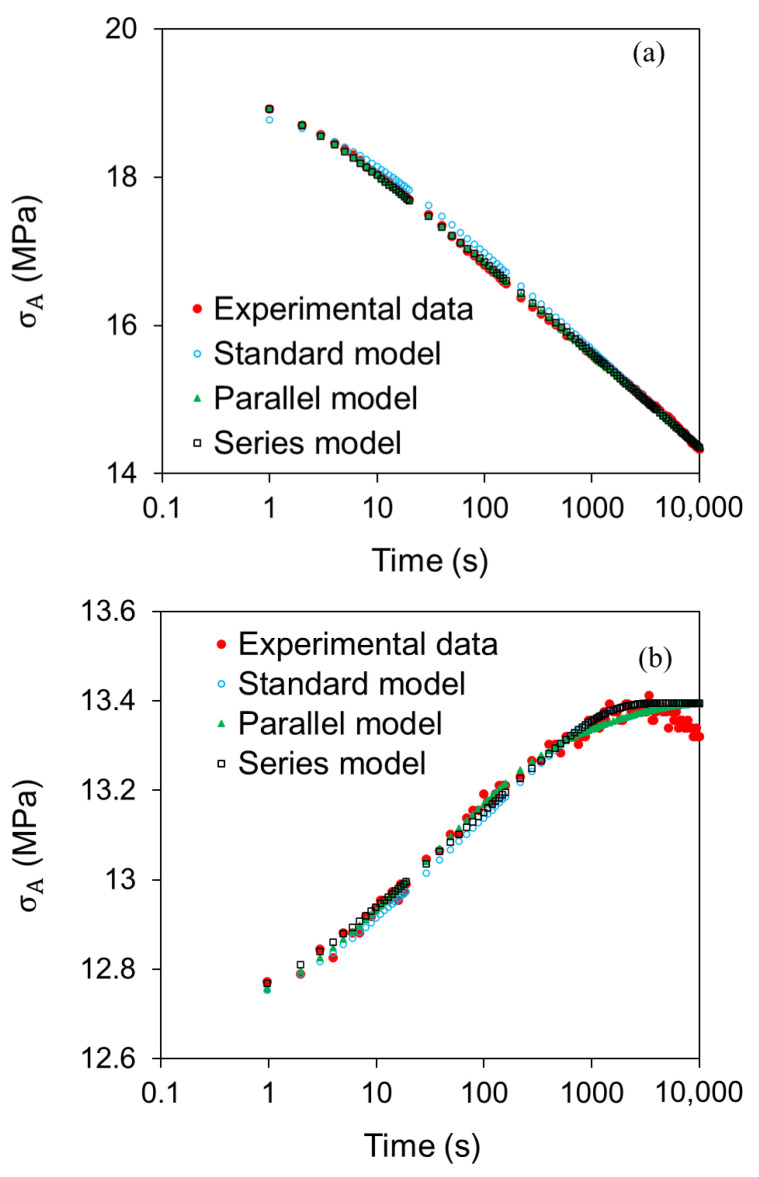
Sample curves for simulation of stress response in the relaxation and recovery phases using the three models in Figure 3, taken from RR test data at stroke of 2.24 mm of HDPE-a: (**a**) for the relaxation phase; (**b**) for the recovery phase.

Sample curves generated from the above CRR approach for the three models in Figure 3 are presented in Figure 7, for HDPE-a at the stroke of 2.24 mm. Figure 7a indicates that the curves generated by the Parallel and the Series models are close to the experimental data. For the standard model, although a pretty good agreement was achieved with the experimental curve, some deviation is shown in the section from 1 to 1000 s. Nevertheless, these results are consistent with those reported in the literature [17,34,47].

Figure 7b indicates that all simulation curves mimicked reasonably well the experimental curve in the recovery phase before the maximum stress is reached, but failed to regenerate a small stress drop before the end of the recovery phase. The stress drops in the recovery phase were also reported in the literature. Kitagawa et al. first observed this stress drop and regarded the drop as an “anomalous” phenomenon [48]. Drozdov et al. reported this stress drop as an “unusual” stress response and suggested that accurate modeling of this behavior remains unresolved [22]. Figure 7b shows that the three models considered here also failed to regenerate this stress drop phenomenon. Even though the stress drop during the recovery phase could not be simulated using the models considered in this study, the maximum difference between the experimental data and data generated by the models in the relaxation phase, apart from the very first relaxation phase that did not go through the stress recovery process, is 0.127 MPa for the Series model and 0.116 MPa for the Parallel model. Such a difference is slightly better than the difference reported in the literature, e.g., 0.17 MPa [44], 0.3 MPa [34], and about 1 MPa [49,50]. In view that the Parallel and the Series models show similar closeness in simulating the experimental data, with the former being slightly better than the latter, the Parallel model will be used in the second case study to determine activation energies for HDPE-a.

**Figure 8:**
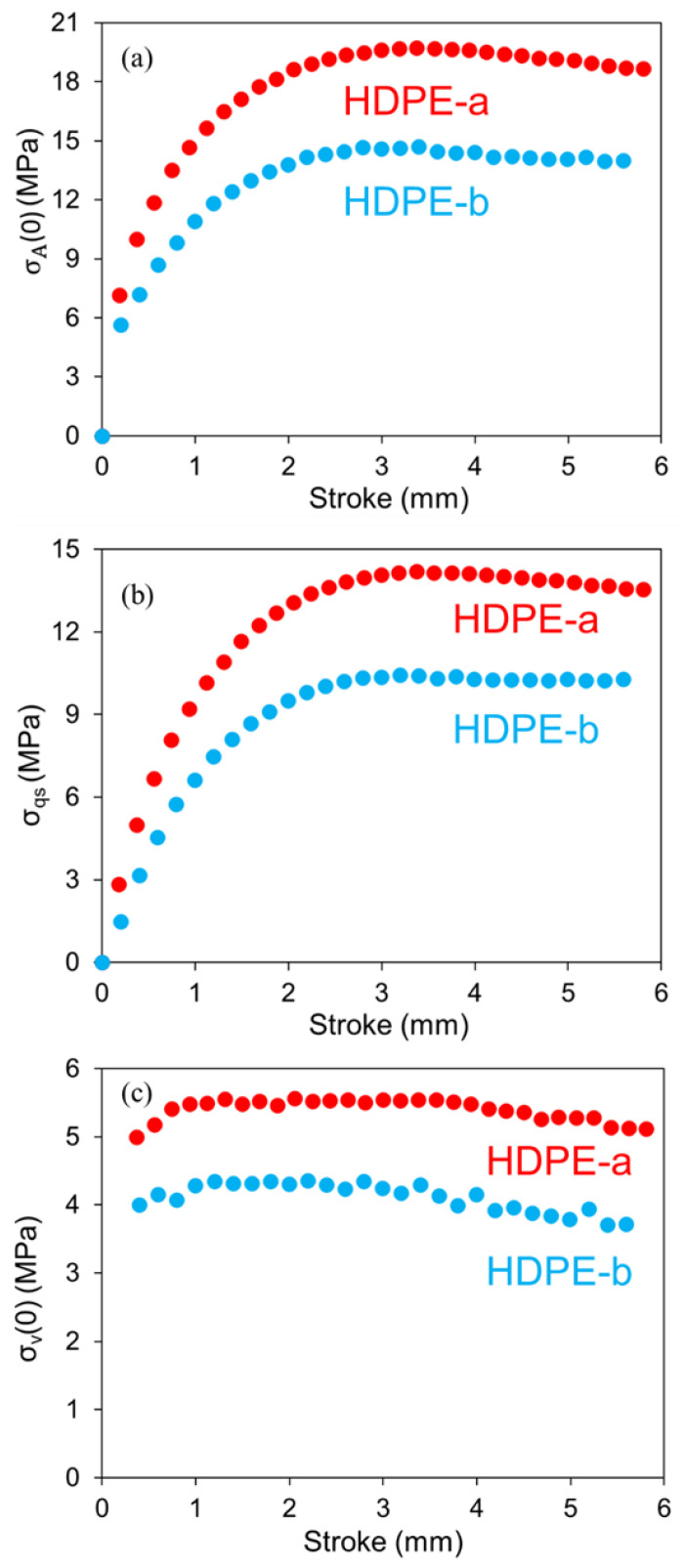
Comparison of stress responses at the beginning of the relaxation phases for the two HDPEs at 294 K: (**a**) $\sigma_{A}\left(0\right)$; (**b**) $\sigma_{\mathrm{qs}}$; (**c**) $\sigma_{v}\left(0\right)$.

Figure 8 compares stress responses to deformation in the RR tests for the 2 HDPEs. Figure 8a depicts the applied stress as a function of stroke at the beginning of the relaxation phase, $\sigma_{A}\left(0\right)$, which indicates that $\sigma_{A}\left(0\right)$ for HDPE-a is higher than that for HDPE-b. The corresponding $\sigma_{\mathrm{qs}}$ and $\sigma_{v}\left(0\right)$ values are presented in Figure 8b,c, respectively. Qualitative difference of the two HDPEs in these stress responses to deformation is expected, but further study is needed using PE of clear difference in the material characteristics, such as molecular weight and its distribution and side branch length, its distribution and density, would be needed to quantify the difference among these stress responses.

**Figure 9:**
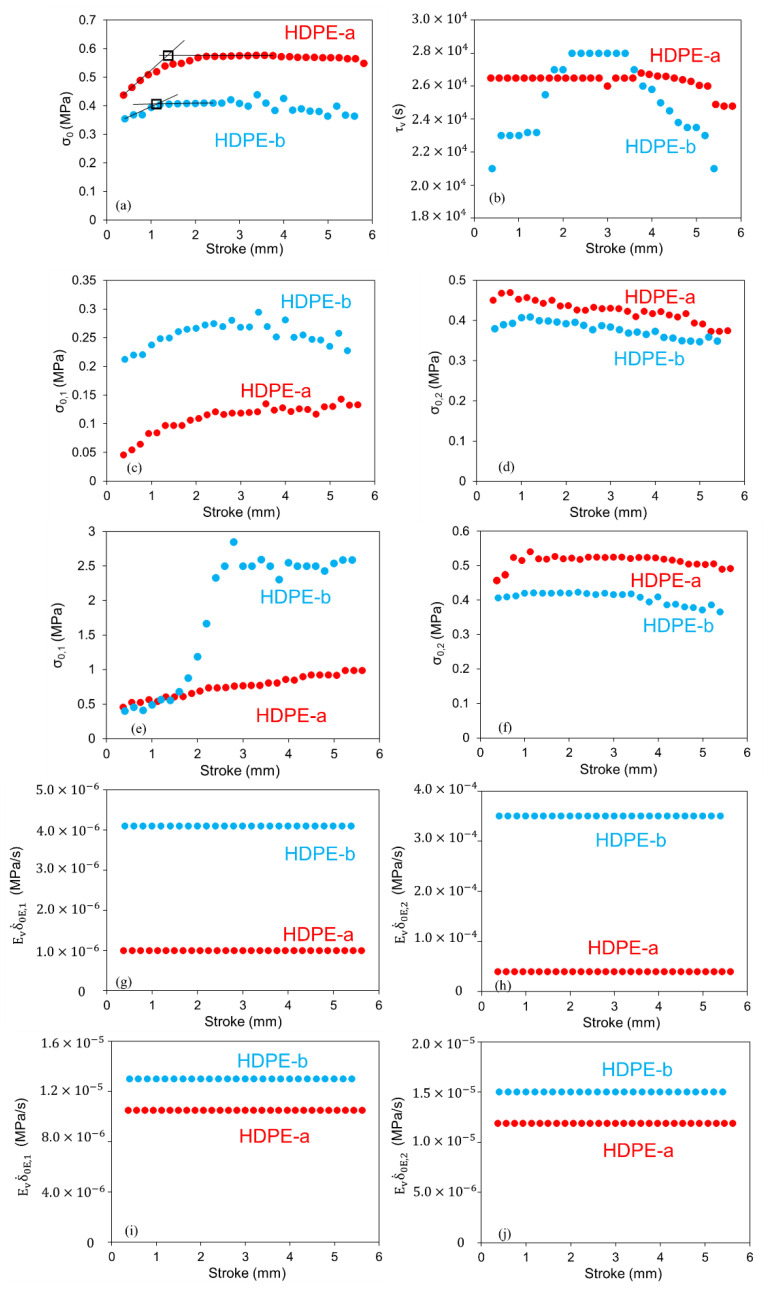
Comparison of model parameters in Figure 9, to simulate stress variation of HDPE-a and HDPE-b as functions of time in the relaxation phases at 294 K: (**a**) ${\;\sigma }_{0}$ for the standard model; (**b**) $\tau_{v}$ for the standard model; (**c**) ${\;\sigma }_{0,1}$ for the Parallel model; (**d**) $\sigma_{0,2}$ for the Parallel model; (**e**) ${\;\sigma }_{0,1}$ for the Series model; (**f**) $\sigma_{0,2}$ for the Series model; (**g**) $E_{v}{\dot{\delta }}_{0E,1}$ for the Parallel model; (**h**) $E_{v}{\dot{\delta }}_{0E,2}$ for the Parallel model; (**i**) $E_{v}{\dot{\delta }}_{0E,1}$ for the Series model; (**j**) $E_{v}{\dot{\delta }}_{0E,2}$ for the Series model.

Values for parameters in viscous branches of the three models in Figure 3 are summarized in Figure 9. For the standard model, as shown in Figure 9a,b, a critical stroke can be detected using the change of $\sigma_{0}$ values. However, further study is needed to examine the influence of allowing the $\tau_{v}$ change on the critical stroke value and whether the critical stroke indicates the change of mechanisms involved in the deformation process [17]. For the Parallel model, Figure 9c,d shows changes of $\sigma_{0,1}$ and $\sigma_{0,2}$, respectively, in the relaxation phase as functions of stroke. Unlike Figure 9a for the standard model, none of the curves in Figure 9c,d shows a clear transition of the trend line that occurs at a common stroke. A similar phenomenon is shown in, Figure 9e,f for the Series model. In addition, Figure 9e indicates a strong change in the trend line for HDPE-b, but not for HDPE-a. Therefore, the critical stroke detected using the standard model, and a critical stroke reported in the past using models with a single dashpot [17,31,51], may need further investigation.

Figure 9g–j indicates that values for $E_{v}{\dot{\delta }}_{0E,1}$ and $E_{v}{\dot{\delta }}_{0E,2}$ in both Parallel and the Series models can maintain constant for the entire RR test. Therefore, a constant value could be chosen for the $E_{v}$ value for each HDPE, and the difference between the corresponding $E_{v}{\dot{\delta }}_{0E,1}$ and $E_{v}{\dot{\delta }}_{0E,2}$ comes from the difference in the ${\dot{\delta }}_{0E,1}$ and ${\dot{\delta }}_{0E,2}$ values. However, in view that neither the Parallel nor the Series model could simulate the stress drop in the recovery phase, as shown in Figure 7b, determination of the $E_{v}$ value was not pursued here. Rather, a study is being conducted when this manuscript is prepared, to develop a model that could mimic the stress drop in the recovery phase, for which the $E_{v}$ value will be determined in the future.

### 5.2. Case Study 2: Determination of Activation Energies for the Eyring’s Model

**Figure 10:**
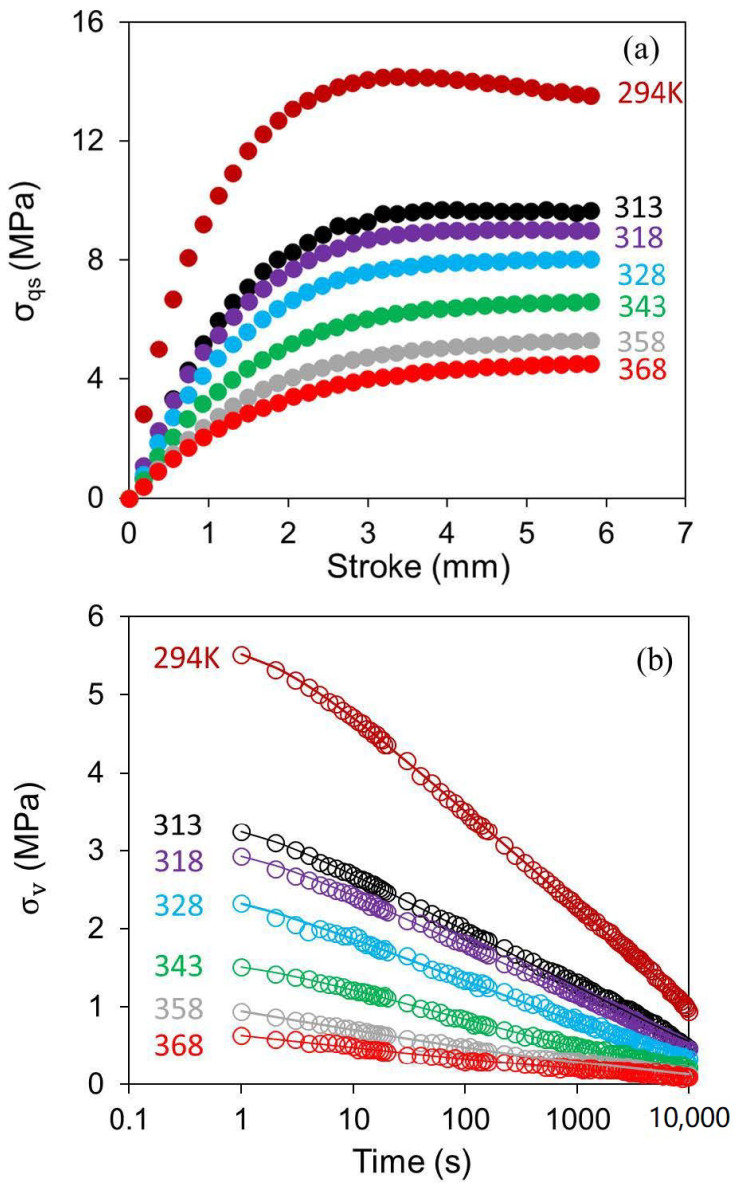
Summary of $\sigma_{\mathrm{qs}}$ (**a**) and $\sigma_{v}$ (**b**) as a function of stroke and time, respectively, at different temperatures ($\sigma_{v}$ was taken from relaxation at the stroke of 3.75 mm at each temperature).

Determination of activation energies was based on RR test results at different temperatures, and analyzed using the Parallel model. Based on Eyring’s law [52], the reference stroke rate (${\dot{\delta }}_{0E,i},\;i=1,\;2$) for the dashpot can be expressed as:

(10) $${\dot{\delta }}_{0E,1}={\;\dot{e}}_{0,1}\exp\left(-\Delta H_{1}\;/\;\mathrm{kT}\right)$$

(11) $${\dot{\delta }}_{0E,2}={\dot{e}}_{0,2}\exp\left(-\Delta H_{2}\;/\;\mathrm{kT}\right)$$

where ${\dot{e}}_{0,i}$ and $\Delta H_{i}$, with i = 1, 2, are the pre-exponential factor and the activation energies, respectively, for the Eyring’s process i, k the Boltzmann’s constant, and T temperature in K. Values for ${\dot{\delta }}_{0E,1}$ and ${\dot{\delta }}_{0E,2}$ at different temperatures were determined by fitting the RR test data in the relaxation phase and the last three points in the prior loading phase. To determine the activation energies, the stroke function of $\sigma_{\mathrm{qs}}$ was first determined using the RR test results at different temperatures, based on the standard model, as shown in Figure 10a. Equation (8) was used for the simulation of relaxation phase and the last three data points in the loading phase before the relaxation phase. Equation (8) was first applied to fit the relaxation phase, to determine values for $E_{v}{\dot{\delta }}_{0E,1}$,${\;E}_{v}{\dot{\delta }}_{0E,2}$, $\sigma_{0,1}$, $\sigma_{0,2}$, and then values for $E_{v}$, ${\dot{\delta }}_{0E,1}$ and ${\dot{\delta }}_{0E,2}$ were determined by fitting the last three data points in the loading phase prior to the relaxation phase. This part of curve fitting was based on the assumption that $E_{v}$, $\sigma_{0,1}$, $\sigma_{0,2}$, ${\dot{\delta }}_{0E,1}$, and ${\dot{\delta }}_{0E,2}$ at the end of the loading phase, before the relaxation, remained constant, with their values to be the same as the corresponding values in the relaxation phase. Since ${\dot{\delta }}_{A}$ was 0 in the relaxation phase and 0.0167 mm/s for the last three data points in the loading phase, with values for $\sigma_{0,1}$, $\sigma_{0,2}$, $E_{v}{\dot{\delta }}_{0E,1}$, and $E_{v}{\dot{\delta }}_{0E,2}$ determined in the fitting process for data in the relaxation phase, using the parallel model described in Section 5.1, values for $E_{v}$, ${\dot{\delta }}_{0E,1}$ and ${\dot{\delta }}_{0E,2}$ were then adjusted to fit the last three data points in the loading phase.

As shown in Figure 10a, $\sigma_{\mathrm{qs}}$ values decrease with the increase of the temperatures. Sample curves in the relaxation phase, from simulation and experiments, are shown in Figure 10b in which the open circles are the experimental data and the solid lines from the simulation. These sample curves depict a good agreement between simulation using the Parallel model and the experimental data, with the maximum difference of 0.125 MPa in the stress value.

**Figure 11:**
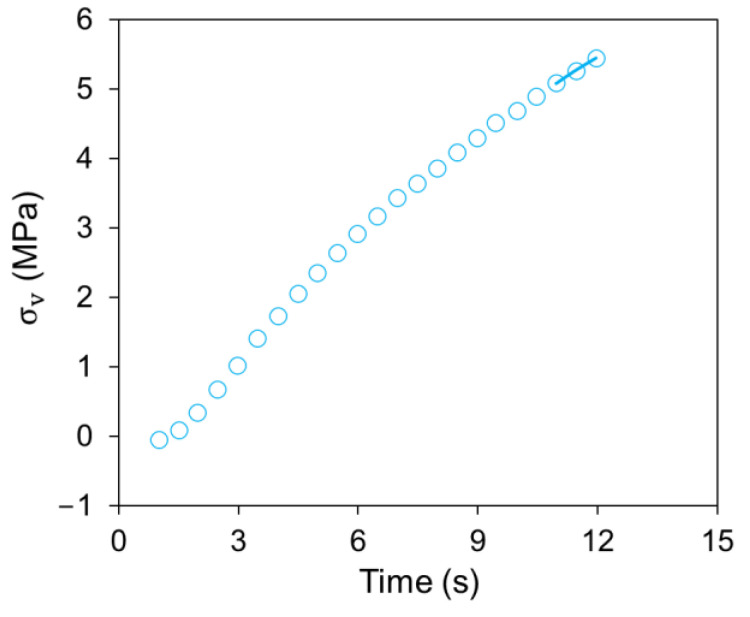
A sample curve of $\sigma_{v}$ versus time in the loading phase between recovery and relaxation, and the fitting line for the last three points based on the Parallel model.

**Figure 12:**
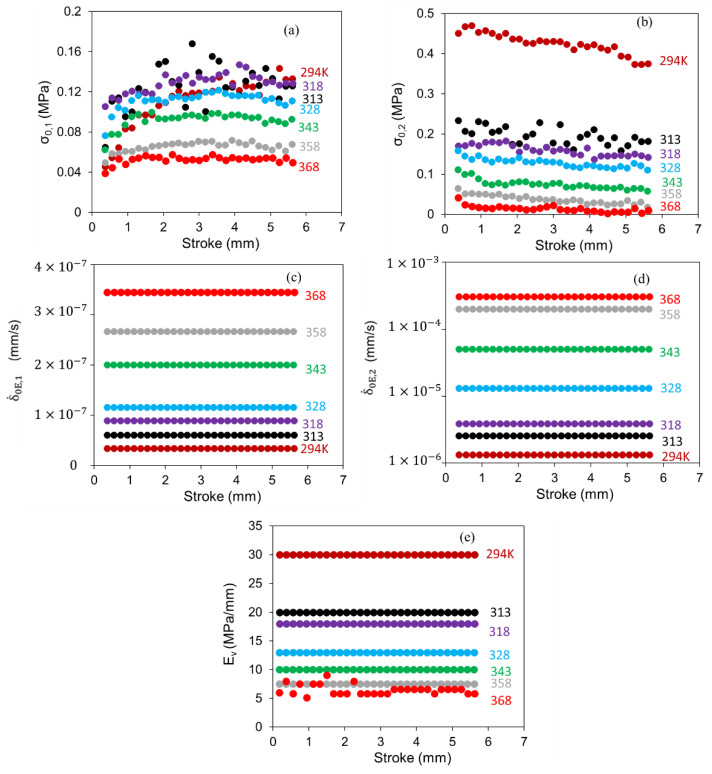
Stroke dependence of parameters used in the Parallel model to simulate the stress response in the relaxation phase at different temperatures: (**a**) $\sigma_{0,1}$; (**b**) $\sigma_{0,2}$; (**c**) ${\dot{\delta }}_{0E,1}$; (**d**) ${\dot{\delta }}_{0E,2}$; (**e**) $E_{v}$.

Figure 11 shows a sample of the simulation curve for the last three data points in the loading phase and all experimental data for the loading phase. The maximum difference in the stress value for all fittings conducted in this case study was 0.069 MPa. Figure 12 summarizes the value for $\sigma_{0,1}$, $\sigma_{0,2}$, ${\dot{\delta }}_{0E,1}$, ${\dot{\delta }}_{0E,2}$, and $E_{v}$ as a function of stroke using the above process. The figure suggests that values for ${\dot{\delta }}_{0E,1}$, ${\dot{\delta }}_{0E,2}$, and E_v_ show little dependence on the stroke, but values for $\sigma_{0,1}$ and $\sigma_{0,2}$ did show some variations with the change of stroke, though the extent of variation decreased with the temperature increase. Figure 12 also suggests that similar to Figure 9c,d, σ_0,1_ and $\sigma_{0,2}$ determined using the Parallel model did not show any clear transition in their dependence on the stroke variation.

**Figure 13:**
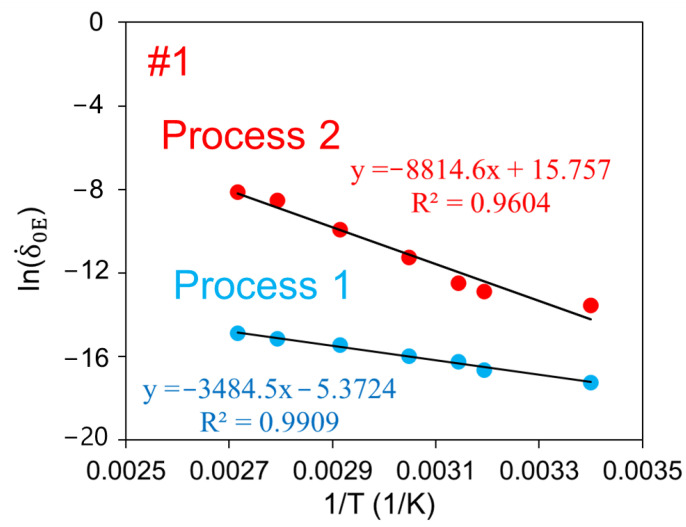
Plot of $\ln\left({\dot{\delta }}_{0E}\right)$ versus 1/T for the Parallel model, and the corresponding equations for the linear curve fitting.

Figure 13 presents the plots of ln(${\dot{\delta }}_{0E,1}$) and ln(${\dot{\delta }}_{0E,2}$) as a function of 1/T, where T is temperature in K. The figure shows that slopes of data points for processes 1 and 2, based on the linear curve fitting, are −3485 and −8815, respectively. Based on Equations (10) and (11), the corresponding activation energies for processes 1 and 2 are 28.96 and 73.25 kJ/mol, respectively, which can be used to quantify the energy barriers for relaxation introduced in the RR test. These values are in the same order of magnitude as those reported in the literature, such as Wilhelm et al. [53] who used creep test data for PE100 pipe resin and determined the activation energy for one Eyring’s process to be 30 kJ/mol and Truss et al. [54] who used torsion test data for HDPE with the density of 0.972 g/cm^3^ and determined the activation energies for yielding using two Eyring’s process connected in parallel to be 243 and 100 kJ/mol.

As suggested by André and Cruz-Pinto [55], in addition to the dependence on materials, different loading modes may also yield different values for the activation energies. Therefore, a further study on the effect of the loading mode on the activation energy will be conducted based on the principle of the RR test presented here.

## 6. Conclusions

A novel RR test was developed, which contains multiple cycles with six stages in one cycle of the test. The test was designed to separate the viscous stress response to deformation from the quasi-static counterpart. The study discovered that a commonly observed hysteresis loop from loading-unloading of polymeric materials is hardly visible between unloading before the recovery phase and the initial loading after the recovery phase in the RR test. Three models were examined to explore their feasibility to analyze the RR test results. It was found that the standard model could not mimic closely the stress drop during the entire relaxation process of 10,000 s, but, the Parallel and the Series models could. It was found that the standard model could determine the long-term performance of polyethylene and reveal a transition point for the onset of plastic deformation in the crystalline phase. However, none of the three models was able to generate a stress drop in the recovery phase after the maximum point which was shown in the experimental data.

The viscous and quasi-static stress responses for two HDPEs were characterized using the RR test based on the three models for the data analysis. It was found that the RR test has the advantage of determining the total stiffness of the materials at different deformation levels, which can be applied to the evaluation of PE’s mechanical performance. The study shows that the RR test provides a data set that can be used to evaluate the suitability of spring-dashpot models for characterizing the time-dependent and time-independent mechanical performance of PE, and the possibility of determining the activation energy for deformation in the stress relaxation mode. A study is being conducted when this manuscript is prepared, to develop a suitable model that can mimic the complex stress response to deformation introduced in the RR test, and determine the corresponding activation energy for deformation introduced in the RR test.

## Figures and Tables

**Table 1:** Characteristics of HDPE-a and HDPE-b used in this study.

Properties	Test Method	Units	HDPE-a	HDPE-b
Density	ASTM D792	g/cm^3^	0.949	0.945
Tensile Strength @ Yield	ASTM D638	MPa	24.1	22.5
Ultimate Elongation	ASTM D638	%	500	850
SCG PENT	ASTM F1473	h	>10,000	>100

## Data Availability

The data supporting the findings described in this manuscript are available from the corresponding authors upon request.
